# What is impact of nonsteroidal anti-inflammatory drugs in the prevention of post-endoscopic retrograde cholangiopancreatography pancreatitis: a meta-analysis of randomized controlled trials

**DOI:** 10.1186/s12876-018-0837-4

**Published:** 2018-07-04

**Authors:** Yunxiao Lyu, Yunxiao Cheng, Bin Wang, Yueming Xu, Weibing Du

**Affiliations:** Department of General Surgery, Dongyang people’s Hospital, Dongyang, 322100 Zhejiang Province China

**Keywords:** NSAIDs, Indomethacin, Diclofenac, ERCP, Pancreatitis, Meta-analysis

## Abstract

**Background:**

Recently, although studies have investigated the role of NSAIDs in the prevention of post-endoscopic retrograde cholangiopancreatography pancreatitis (PEP), selection of the ideal drug, the time and route of its administration for the appropriate population remain controversial.

**Methods:**

A systematic search was done in sources including PubMed, Embase, Web of Science, the Cochrane Library Central, and ClinicalTrials.gov from from August 1, 1990 to August 1, 2017. Randomized controlled trials comparing the prophylactic use of NSAIDs versus a placebo were included. Statistical analysis was performed using the RevMan 5.3 software to assess the outcomes.

**Results:**

A total of 21 randomized controlled trials were included in the meta-analysis. Our study showed that NSAIDs significantly reduced the incidence of PEP (RR, 0.61, 95%CI,0.52–0.72; *p* < 0.00001). The analysis showed that indomethacin administration post-ERCP (RR, 0.47; 95% CI, 0.31–0.70; *p* = 0.0002) appeared to be more effective in preventing PEP than indomethacin administration pre-ERCP (RR, 0.59; 95% CI, 0.45–0.79; *P* = 0.0003), but there was no significant difference between the high-risk and average-risk population(*p* = 0.13). In the diclofenac group, it was noted that administration of diclofenac pre-ERCP (RR, 0.32; 95% CI, 0.16–0.63; *p* = 0.001) was more effective than that in post-ERCP (RR, 0.65; 95% CI, 0.27–1.599; *p* = 0.35). The relative risk of PEP was 0.63 (95% CI, 0.27–1.50; *p* = 0.30) in high-risk patients and 0.41 (95% CI, 0.17–0.98; *p* = 0.02) in average-risk patients. With regard to the route of administration, PEP decreased significantly only in patients receiving the drug rectally (RR, 0.53; 95% CI, 0.44–0.63; *p* < 0.00001), but not for those who received intramuscularly (RR, 0.74; 95% CI, 0.47–1.17; *p* = 0.20), intravenously (RR, 0.97; 95% CI, 0.51–1.83; *p* = 0.93), and orally (RR = 0.88; 95% CI, 0.55–0.1.43; *p* = 0.62).

**Conclusions:**

Rectal administration of NSAIDs (both indomethacin and diclofenac) was effective in preventing PEP in unselected patients. A single dose of indomethacin after ERCP might be effective in preventing PEP in both high-risk and average-risk patients. However, diclofenac administered rectally before ERCP might be protective against PEP in high-risk patients compared to a placebo. However, more high quality head-to-head RCTs are required.

## Background

With the continuous improvement of endoscopic instruments and technical means, endoscopic retrograde cholangiopancreatography (ERCP) has gained more attention from clinicians for the diagnosis and treatment of biliary and pancreatic diseases. ERCP via its minimally invasive benefit in the diagnosis of biliary and pancreatic diseases is challenged by a higher potential for serious complications than any other standard endoscopic technique. Frequent complications after ERCP include pancreatitis, postoperative bleeding, and gastrointestinal perforation. The most frequent of these is pancreatitis. Due to the different group characteristics and modes of operation, the incidence of post-ERCP pancreatitis (PEP) is not the same; some studies have reported it to be about 2–4%, while others have reported that it could increase to 8–20% in some high-risk patients, [1]. Most patients with PEP have a mild onset and are cured after minimal treatment. However, some patients develop severe pancreatitis and eventually die. The reported mortality rate is approximately 0.2–0.6% [2, 3]. Thus, more effective prevention methods are still needed. To date, many drugs and endoscopic methods have been investigated to prevent the occurrence of PEP [4–6]. Prophylactic pancreatic duct stenting is considered to effectively prevent PEP in some studies, but its clinical use is limited due to the need for higher technical capacity and cost. Drug classes that have been studied to prevent PEP include nifedipine, nitroglycerin, steroids, protease inhibitors, and somatostatin [7–12]. NSAIDs have been shown to decrease the incidence of PEP. Early studies of randomized controlled trials (RCTs) explored the administration of indomethacin or diclofenac rectally for the prevention of PEP. Subsequent meta-analysis and RCT studies confirm the role of NSAIDs in preventing PEP. Given these previous studies, the European Society of Gastrointestinal Endoscopy (ESGE) and the Japanese Society of Hepato-Biliary-Pancreatic Surgery guidelines recommend routine administration of indomethacin to prevent PEP [13, 14]. However, some recent high-quality RCT studies have shown that NSAIDs may not play a role in reducing the incidence of PEP [15–17]. In different RCT studies, the drug type, route of administration, time of the administration, and study population are not the same, with a lack of a targeted head in the clinical research. At the same time, recent meta-analysis indicated different conclusions about the specific time and route of administration of NSAIDs to prevent PEP. A survey from 29 countries showed a skeptical attitude towards NSAIDs in preventing PEP by a significant percentage of clinicians because of the lack of convincing evidence [18]. Therefore, in order to further clarify the role of NSAIDs in the prevention of PEP, determine the specific drug type, time and route of administration, and its application to the appropriate population, a more systematic, comprehensive, and rigorous evaluation is necessary. In the current study, we collected high-quality RCTs to provide a reliable evidence base for clinical trials of NSAIDs in the prevention of PEP.

## Methods

### Search strategy

Two authors (YX.C.and B.W.) independently conducted a comprehensive search in sources including PubMed, Embase, Web of Science, Cochrane Library Central, and ClinicalTrials.gov from August 1, 1990 to August 1, 2017. English search terms included albeit were not limited to the following: nonsteroidal anti-inflammatory drugs, NSAIDs, diclofenac, indomethacin, post-endoscopic retrograde cholangiopancreatography pancreatitis, post-ERCP pancreatitis, pancreatitis, endoscopic retrograde cholangiopancreatography, and ERCP. The search was limited initially to publications of human RCTs. The references of the articles identified after an initial search were also manually reviewed.

### Inclusion and exclusion criteria

The following inclusive selection criteria were applied: (1) An RCT must compare the incidence of PEP with NSAID and placebo administration or no treatment. (2) The participants must have had a clinical diagnosis of PEP. (3) Studies must report the drug type, route of administration, time of administration, and incidence of PEP in each arm.

We excluded those that (1) were non-RCTs, retrospective studies, review articles, case reports, abstract, editorials, and letters to the editor, (2) published by the same author or agency repeatedly, and (3) had insufficient data on outcome measures of PEP.

### Data extraction

In order to ensure the homogeneity of the extracted data, two authors (YM.W and WB.D) independently extracted the original data in the literature onto a standardized form: the first author, year of publication, country, sample size, types of NSAIDs, drug dose, time of administration, route of administration, and study population. If necessary, we contact the author of the study to obtain the study data. Conflicts in data abstraction were resolved by a consensus, and by referring to the original article.

### Risk of bias asscessment

The authors independently assessed the quality of the literature in accordance with the Cochrane Collaboration Handbook [19]. The scoring system included the following criteria: random sequence generation, allocation concealment, blinding of participants and personnel, blinding the result assessment, incomplete data of the results, selective reporting, and other sources of bias.

Study quality was also assessed with the Jadad scale of randomized controlled trials (RCTs) [20]. Two reviewers (YX.L. and B.W.) independently assessed the quality of the included studies and discrepancies were resolved by discussion in plenum.

### Statistical analysis

All statistical analyses were performed using the Review Manager (RevMan) version 5.3 software (Cochrane Informatics and Knowledge Management Department). Risk ratios (RR) with a 95% confidence interval (CI) were used for dichotomous outcomes. Studies with an I^2^ of 25 to 50% were considered to have low heterogeneity; studies with an I^2^ of 50 to 75% were considered to have moderate heterogeneity; and studies with an I^2^ > 75% were considered to have high heterogeneity.Random effect modelling was applied if the I^2^ > 50%. If not, fixed effect models were constructed. The publication bias was evaluated by χ2 test and funnel plots. The heterogeneity among studies was evaluated by χ2 test. A 2-tailed *P* value of < 0.05 was considered to be statistically significant. We also assessed the potential for publication bias through a visual inspection of a funnel plot asymmetry. The meta-analysis was conducted according to the PRISMA statement.

## Results

### Selected study and characteristics of the trials

**Fig. 1:**
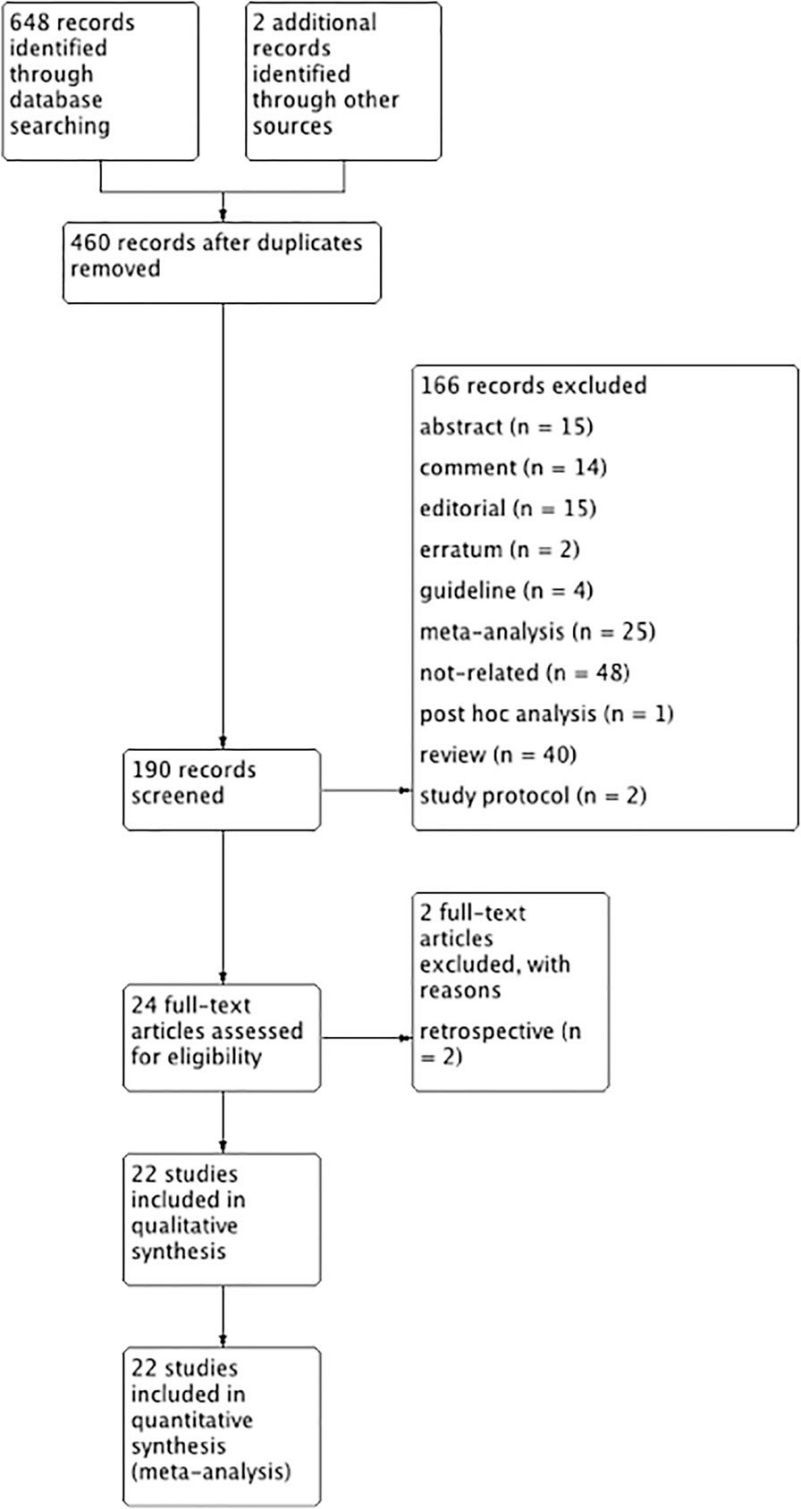
Flow diagram of the published articles evaluated for inclusion in this meta-analysis

Based on our search criteria, we identified 650 papers from the respective search engines, of which 460 duplicate articles were excluded. The remaining 190 studies were retrieved for their titles and abstracts, leaving 24 articles that appeared to meet our selection criteria. From these articles, three were excluded because they were retrospective studies. Finally, 21 RCTs [15–17, 21–38] with 6134 participants were included in the meta-analysis. A detailed flowchart of the selection process is shown in Fig. 1.

The 6134 patients were divided equally into 3082 in the treatment and 3052 into control groups, respectively. Sample sizes ranged from 100 to 665, and the incidence rate of PEP varied from 2.24–16.43%. Diclofenac was used in eight studies [16, 21, 27, 29, 30, 32, 34, 38], indomethacin in 9 studies [15, 17, 22, 24-26, 28, 31, 33, 38], while valdecoxib [23], ketoprofen [37], naproxen [35], and celecoxib [36] were used in one study, respectively. NSAIDs were administered rectally in 14 studies [15–17, 22, 24–31, 33, 35], orally in two studies [36, 38], intravenously in two studies [23, 37], intramuscularly in two studies [21, 32], and intramuscularly and rectally in one study [34]. NSAIDs were administered pre-ERCP in 13 studies [17, 21, 23, 24, 26, 28, 30, 31, 33–38], post-ERCP in six studies [16, 22, 25, 27, 29, 32], during ERCP in one study [15], and pre-ERCP combined with post-ERCP in one study [38]. Six studies evaluated only patients at high-risk for developing PEP [16, 22, 25, 27, 29, 38], whereas 15 studies evaluated patients at average-risk for developing PEP [15, 17, 21, 23, 24, 26–28, 30–36]. The main characteristics of the studies included in this meta-analysis are shown in Table 1.

**Table 1:** Characteristics of studies included in the systematic review

		Sample size			Jadad score
Author Year	Setting	NSAIDs(n)	Placebo(n)	NSAIDs intervention	
Abu-Safieh [21] et al. 2014	Single center	89	93	75 mg diclofenac intramuscular before ERCP	3
Andrade-Dávila [22] et al. 2015	Single center	82	84	100 mg indomethacin rectal immediately after ERCP	3
Bhatia [23] et al. 2011	Single center	121	126	20 mg valdecoxib intravenous at the start of ERCP.	3
Cheon [38] et al. 2007	Single center	105	102	50 mg diclofenac 30–90 min oral before ERCP and 4–6 h after	5
Döbrönte [24] et al. 2012	Single center	130	98	100 mg indomethacin rectal 10 min before ERCP	2
Döbrönte [17] et al. 2014	Multicenter	347	318	100 mg indomethacin rectal 10–15 min before sedo-analgesic premedication	2
Elmunzer [25] et al. 2012	Single center	295	307	100 mg indomethacin rectal immediately after ERCP	5
Hosseini [26] et al. 2016	Single center	100	105	100 mg of indomethacin rectal two hours before the ERCP procedure	3
Kato [36] et al. 2017	Single center	85	85	400 mg celecoxib oral 1 h before ERCP	2
Khoshbaten [27] et al. 2008	Single center	50	50	100 mg diclofenac rectal after ERCP within 1 h	4
LevenickV [15] et al. 2015	Single center	223	226	2 * 50 mg indomethacin rectal during ERCP	5
Lua [16] et al. 2015	Single center	69	75	100 mg diclofenac rectal immediately after ERCP	2
Mansour-GhanaeiV [35] et al. 2016	Multicenter	162	162	500 mg naproxen rectal immediately before ERCP.	3
Montaño LozaV [28] et al. 2007	Single center	75	75	100 mg indomethacin rectal two hours before the procedure	1
Murray [29] et al. 2003	Single center	110	110	100 mg indomethacin rectal 2 h before ERCP	5
Otsuka [30] et al. 2012	Multicenter	51	53	50 mg (25 mg, if body weight < 50 kg) diclofenac rectal 30 min before ERCP	2
Park [32] et al. 2015	Single center	173	170	90 mg diclofenac intramuscular immediately after ERCP	5
Patai [31] et al. 2015	Single center	270	269	100 mg indomethacin rectal within 1 h before ERCP	5
Quadros Ono’frio [37] et al. 2017	Single center	224	223	100 mg ketoprofen intravenous during 20 min, immediately before the procedure,	2
Sotoudehmanesh [33] et al. 2007	Single center	221	221	100 mg indomethacin rectal mmediately before ERCP	4
UÇAR [34] et al. (1) 2016	Single center	50	50	100 mg diclofenac sodium rectal 30–90 min before the procedure.	2
UÇAR [34] et al. (2) 2016	Single center	50	50	75 mg diclofenac sodium IM 30–90 min before the procedure.	2

### Methodological quality and risk of bias

**Fig. 2:**
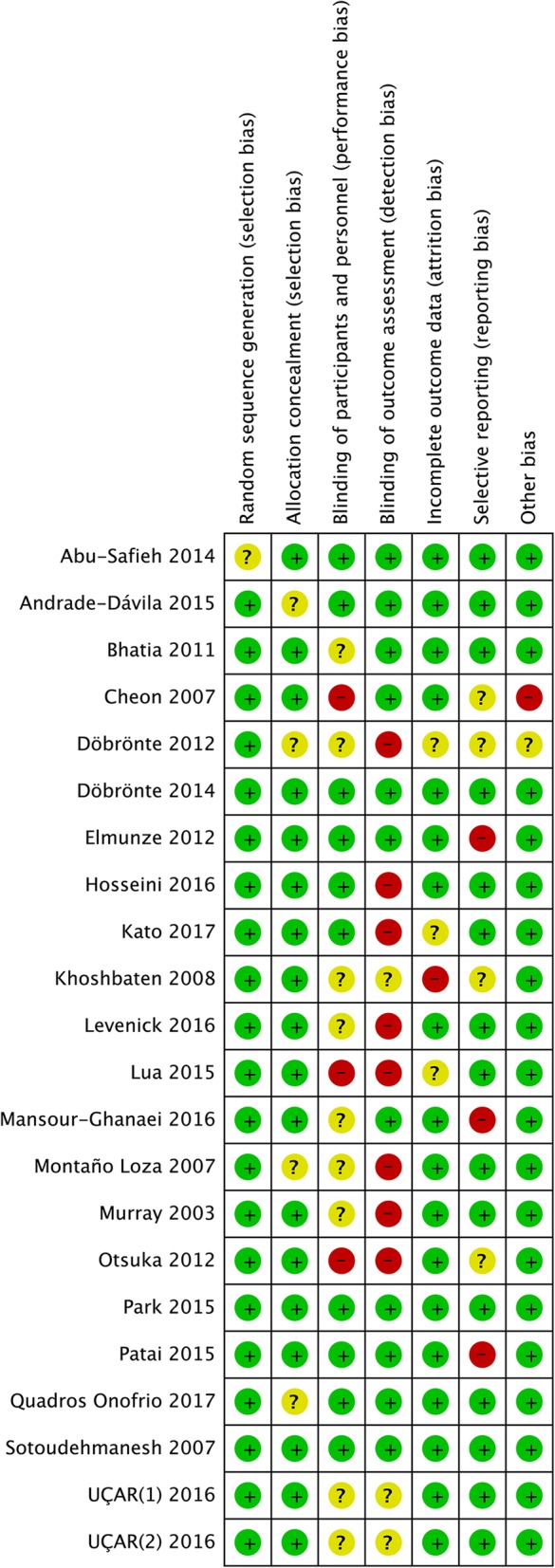
Consensus risk of bias assessment of the included studies. Green, low risk; yellow, unclear; red, high risk

Methodological quality of included studies was evaluated by two investigators (YX.C. and B.W.) using the Cochrane Collaboration tool for assessing the risk of bias. Each trial was given an overall summary assessment of low, unclear, or high risk of bias. Discrepancies in the quality assessment were discussed and resolved by two reviewers (YM.X. and WB.D.). Figure 2 presents an overview of the methodological quality of the studies included in the review.

### Sensitivity analysis

The influence of a single study on the overall meta-analysis estimate was investigated by omitting one study at a time, and the omission of any study made no significant difference, indicating that our results were statistically reliable.

### Risk reduction of PEP

**Fig. 3:**
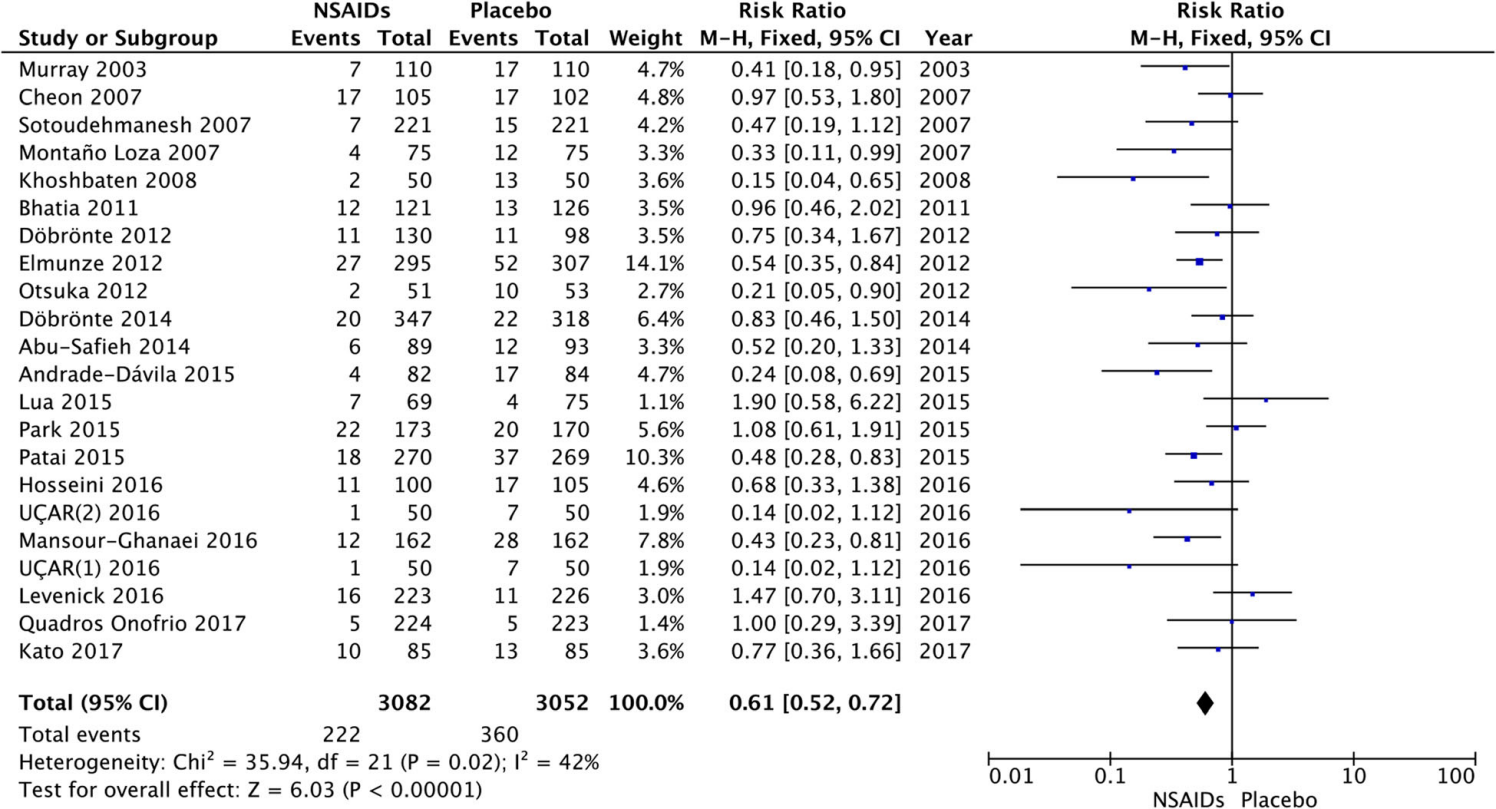
Forest plot of the meta-analysis comparing NSAIDs and placebo for incidence of PEP

We accepted the anthor’s classification stratification of the original studies because the border between studies performed on average-risk or high-risk patients was not well defined. Figure 3 shows the risk of PEP among all the included studies. Because the heterogeneity among these studies was not significant (I^2^ = 42%), we calculated the pooled estimates using the fixed-effects model. The RR of PEP was decreased by NSAIDs to 0.59 (95% CI, 0.51–0.68; *P* < 0.001).

### Type of NSAIDs

**Fig. 4:**
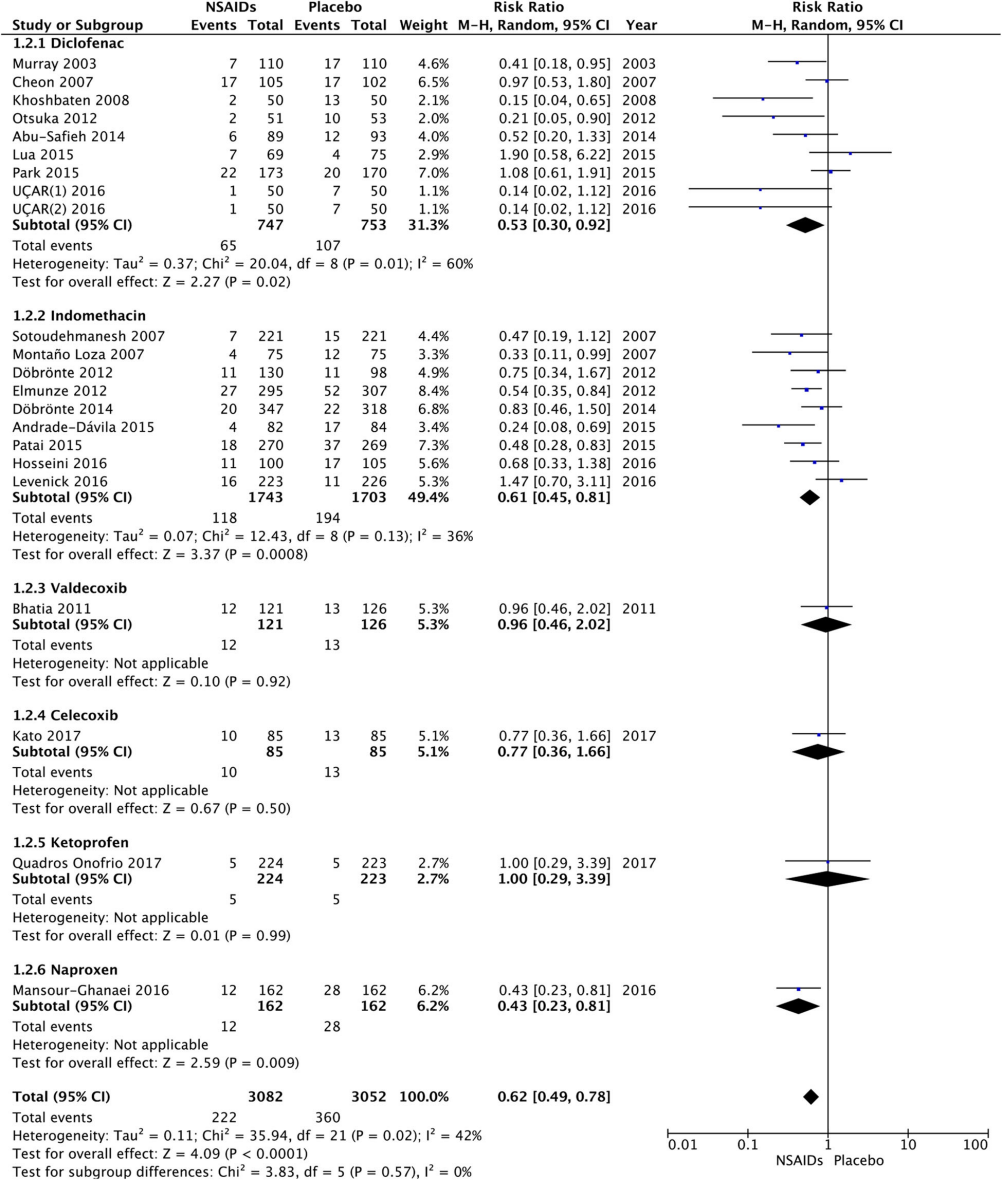
Forest plot of the subgroup meta-analysis of the incidence of PEP with different types of NSAIDs

We calculated the pooled estimates using the fixed-effects model (I^2^ = 42%). Compared with a placebo, the RR of PEP significantly decreased to 0.61 (95% CI, 0.45–0.82; *P* = 0.0009) in the diclofenac group, to 0.60 (95% CI, 0.48–0.74; *P* < 0.00001) in the indomethacin group, and to 0.43 (95% CI, 0.23–0.81; *P* = 0.009) in the naproxen group. The results with the different types of NSAIDs did not differ significantly (*P* = 0.55) (Fig. 4).

### Route of administration

**Fig. 5:**
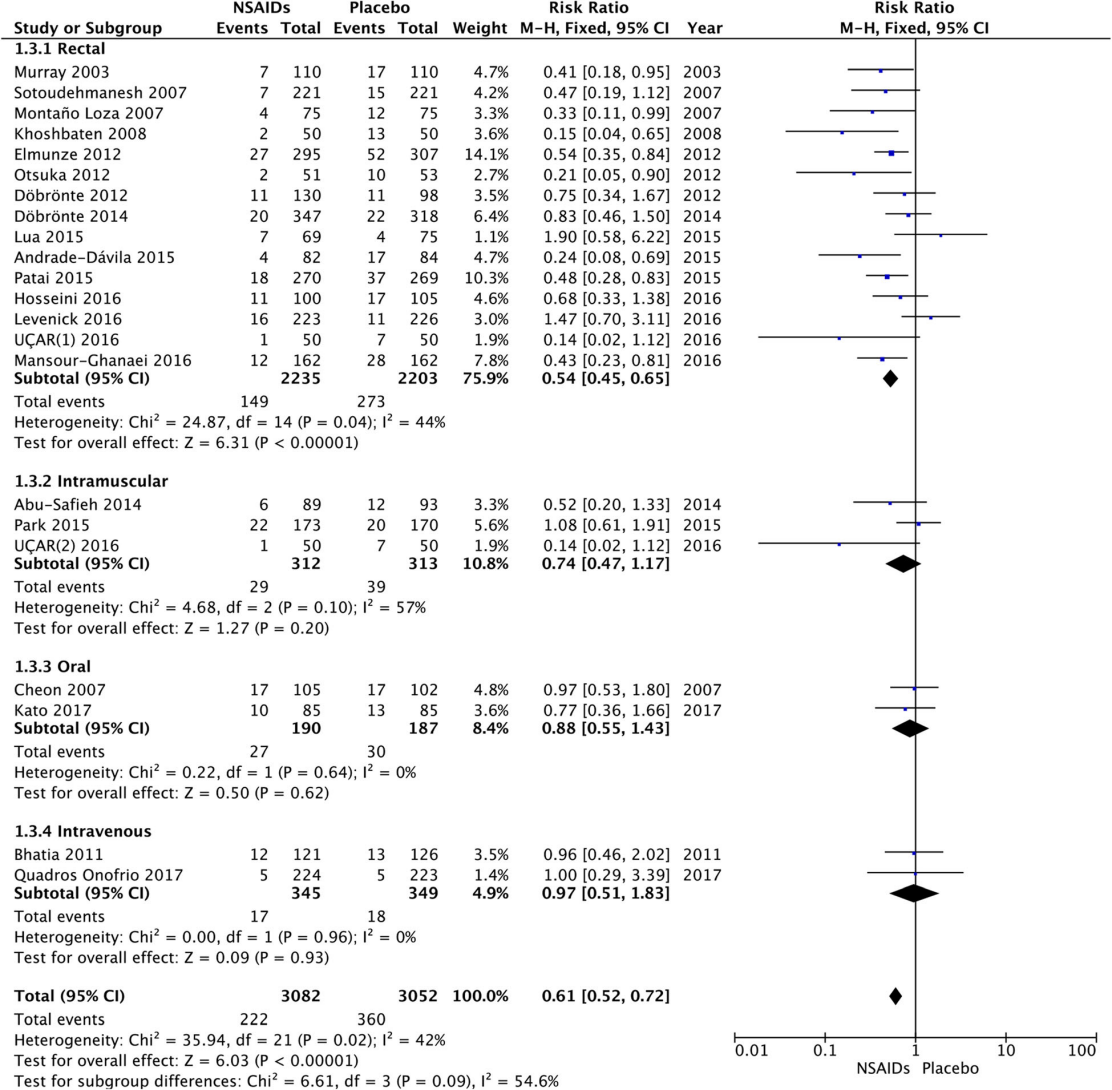
Forest plot of the subgroup meta-analysis of the incidence of PEP based on the route of administration

**Fig. 6:**
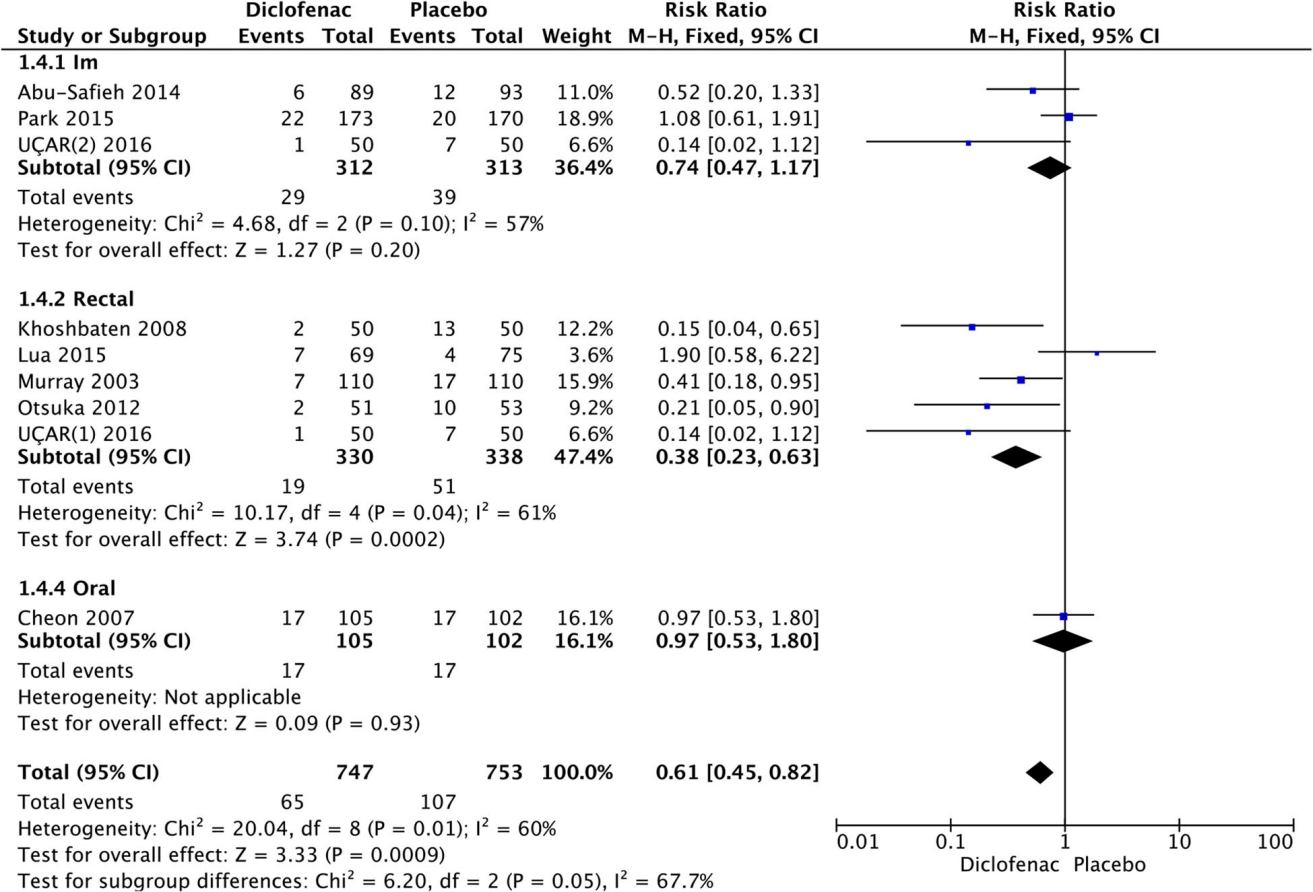
Forest plot of the subgroup meta-analysis of the incidence of PEP based on the route of administration in the diclofenac group

We calculated the pooled estimates using the fixed-effects model (I^2^ = 42%). The route of administration of the NSAIDs was divided into four: rectal, intramuscular, intravenous, and oral. PEP decreased significantly only in patients who received the drug rectally (RR = 0.54; 95% CI, 0.45–0.65; *P* < 0.00001), but did not for those who received the drug intramuscularly (RR = 0.74; 95% CI, 0.47–1.17; *P* = 0.20), intravenously (RR = 0.97; 95% CI, 0.51–1.83; *P* = 0.93), and orally (RR = 0.88; 95% CI, 0.55–0.1.43; *P* = 0.62) (Fig. 5). On comparing the incidence of PEP in the different routes of administration, rectal administration was the only effective route in the diclofenac group (RR = 0.38; 95% CI, 0.23–0.63; *P* = 0.0002) (Fig. 6).

### Time of administration

**Fig. 7:**
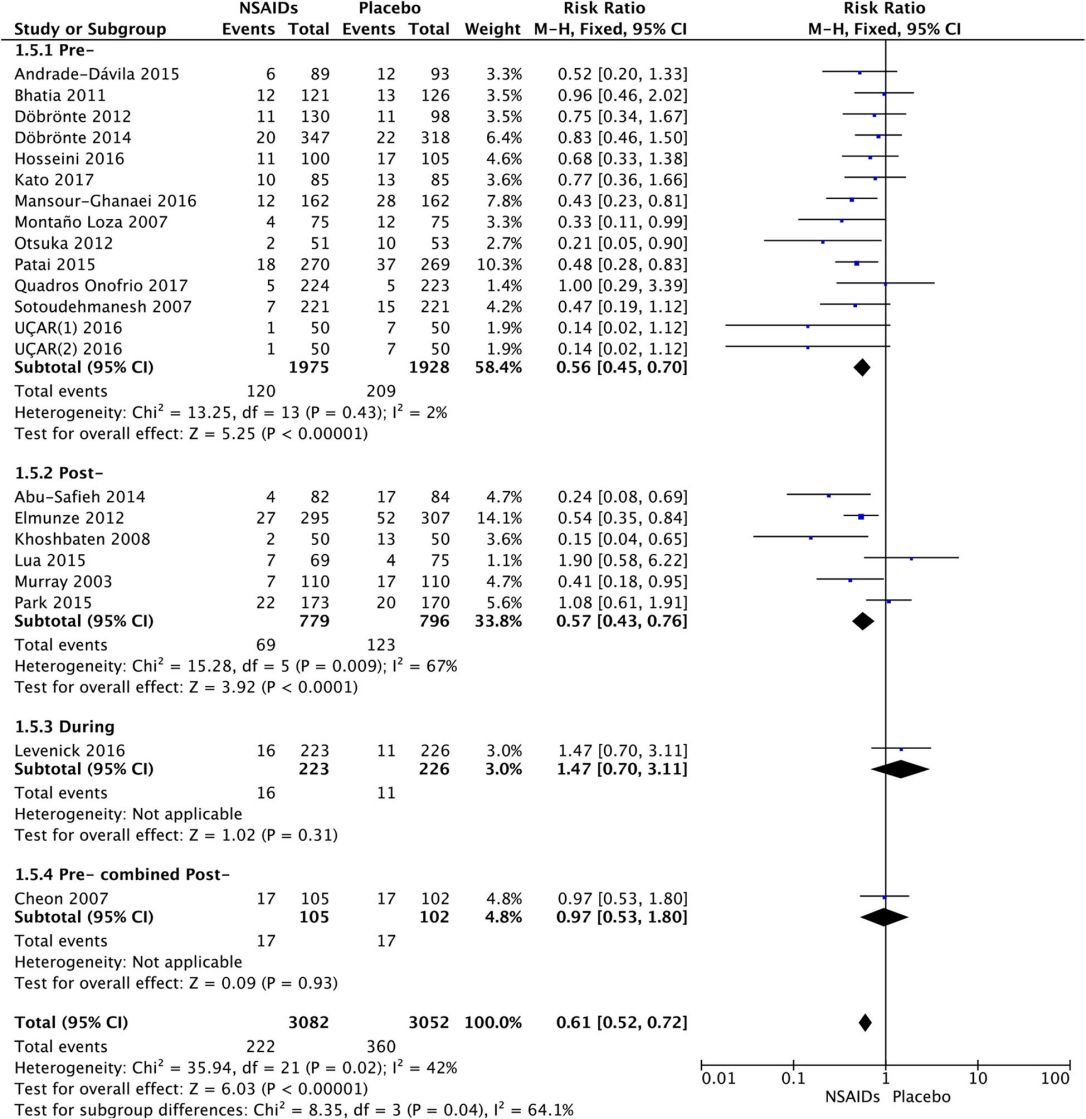
Forest plot of the subgroup meta-analysis of the incidence of PEP based on the time of administration

**Fig. 8:**
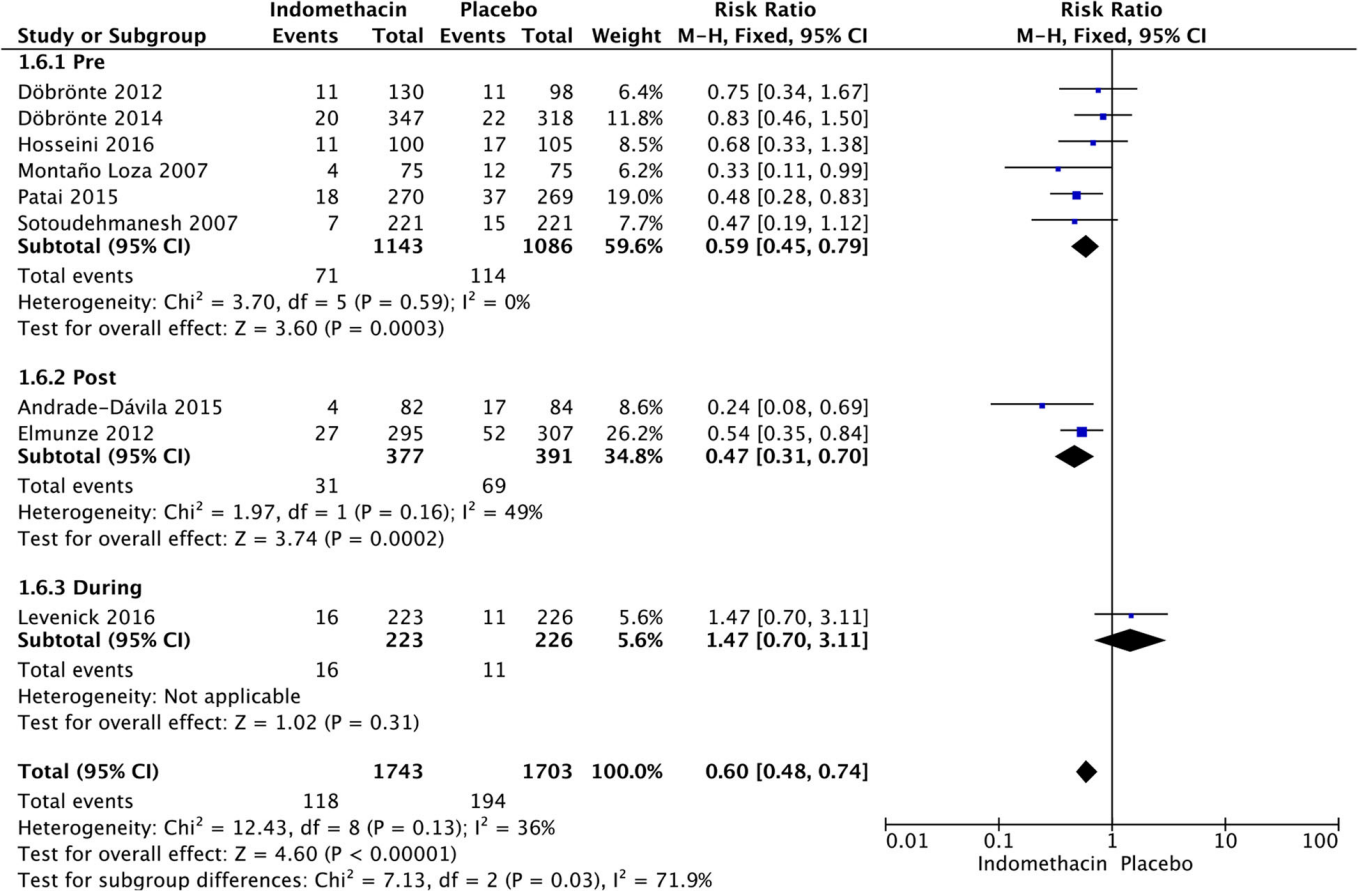
Forest plot of the subgroup meta-analysis of the incidence of PEP based on the time of administration in the indomethacin group

**Fig. 9:**
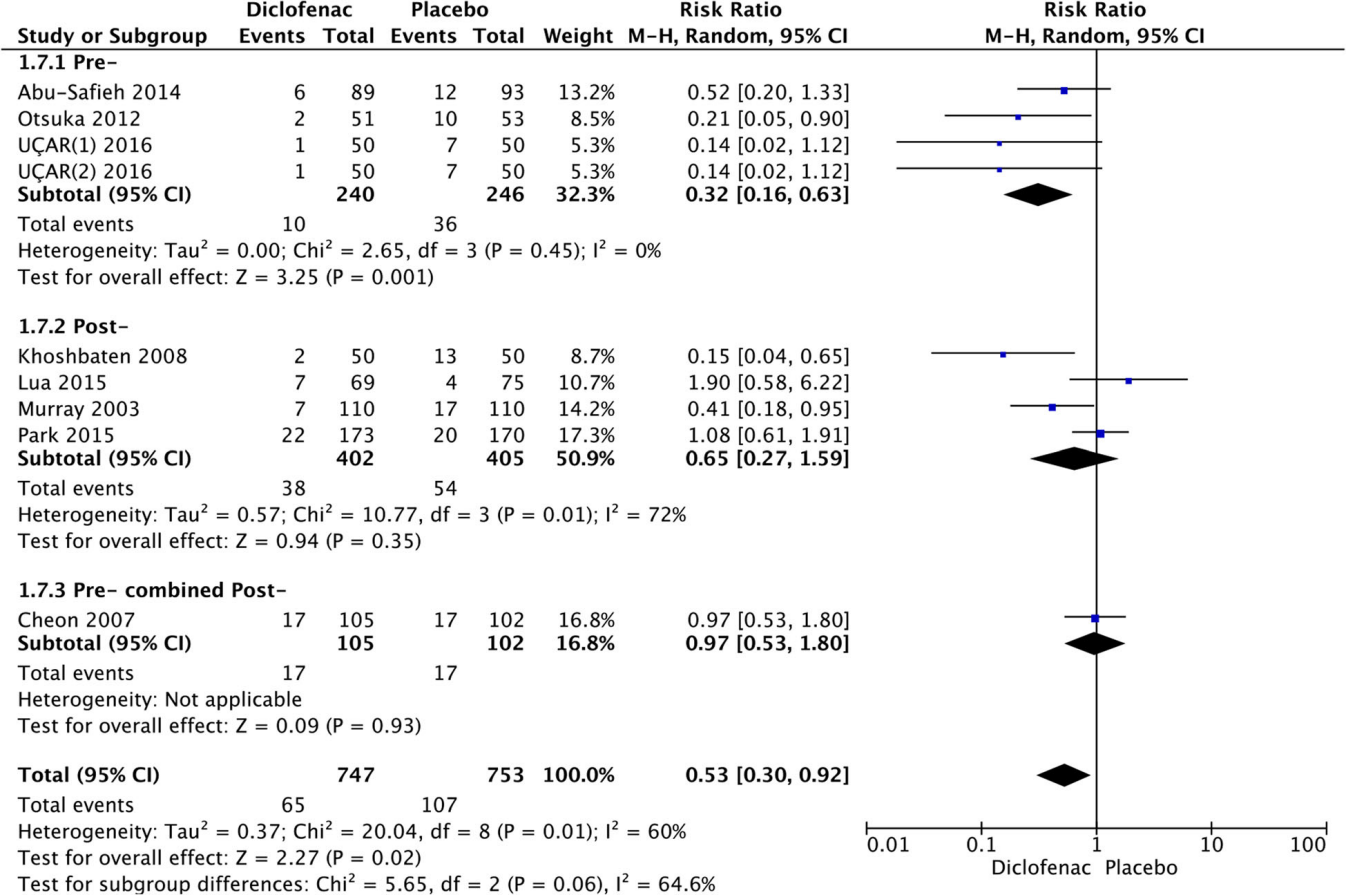
Forest plot of the subgroup meta-analysis of the incidence of PEP based on the time of administration in the diclofenac group

We calculated the pooled estimates using the fixed-effects model (I^2^ = 42%). The efficacy of NSAIDs was compared according to the time of administration, presented in Fig. 7. After stratifying the subgroups by time of administration, NSAIDs administered pre-ERCP (RR = 0.56; 95% CI, 0.45–0.70; *P* < 0.00001) were more effective than those administered post-ERCP (RR = 0.57; 95% CI, 0.43–0.76; *P* < 0.0001) (Fig. 7). The analysis showed that indomethacin administered post-ERCP (RR = 0.47; 95% CI, 0.31–0.70; *P* = 0.0002) appeared to be more effective in preventing PEP than those administered pre-ERCP (RR = 0.59; 95% CI, 0.45–0.79; *P* = 0.0003) (Fig. 8). In the diclofenac group, it was noted that the drug administered pre-ERCP (RR = 0.32; 95% CI, 0.16–0.63; *P* = 0.001) was more effective in preventing PEP than those administered post-ERCP (RR = 0.65; 95% CI, 0.27–1.599; *P* = 0.35) (Fig. 9).

### Average-risk versus high-risk

**Fig. 10:**
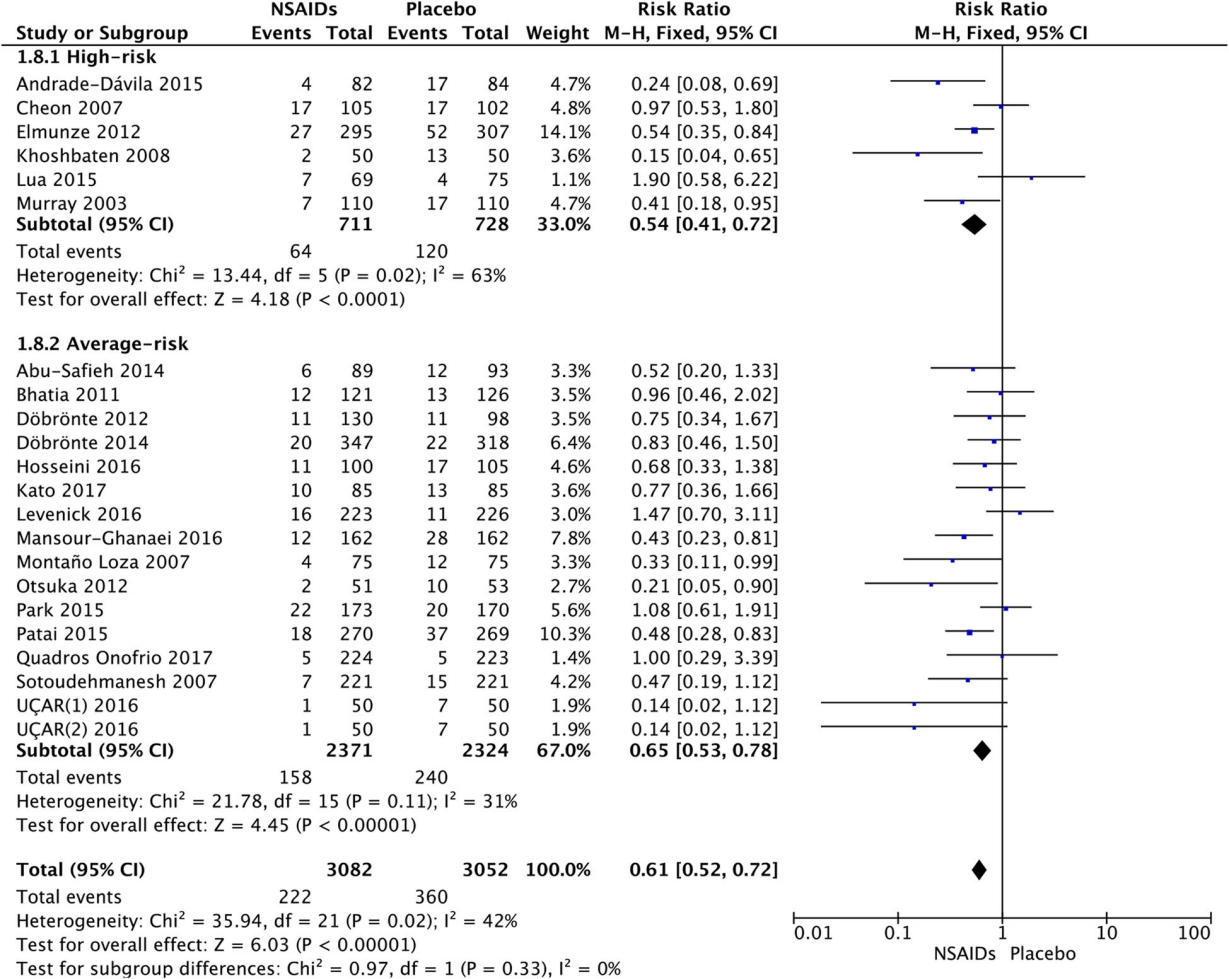
Forest plot of the subgroup meta-analysis of the incidence of PEP by risk

**Fig. 11:**
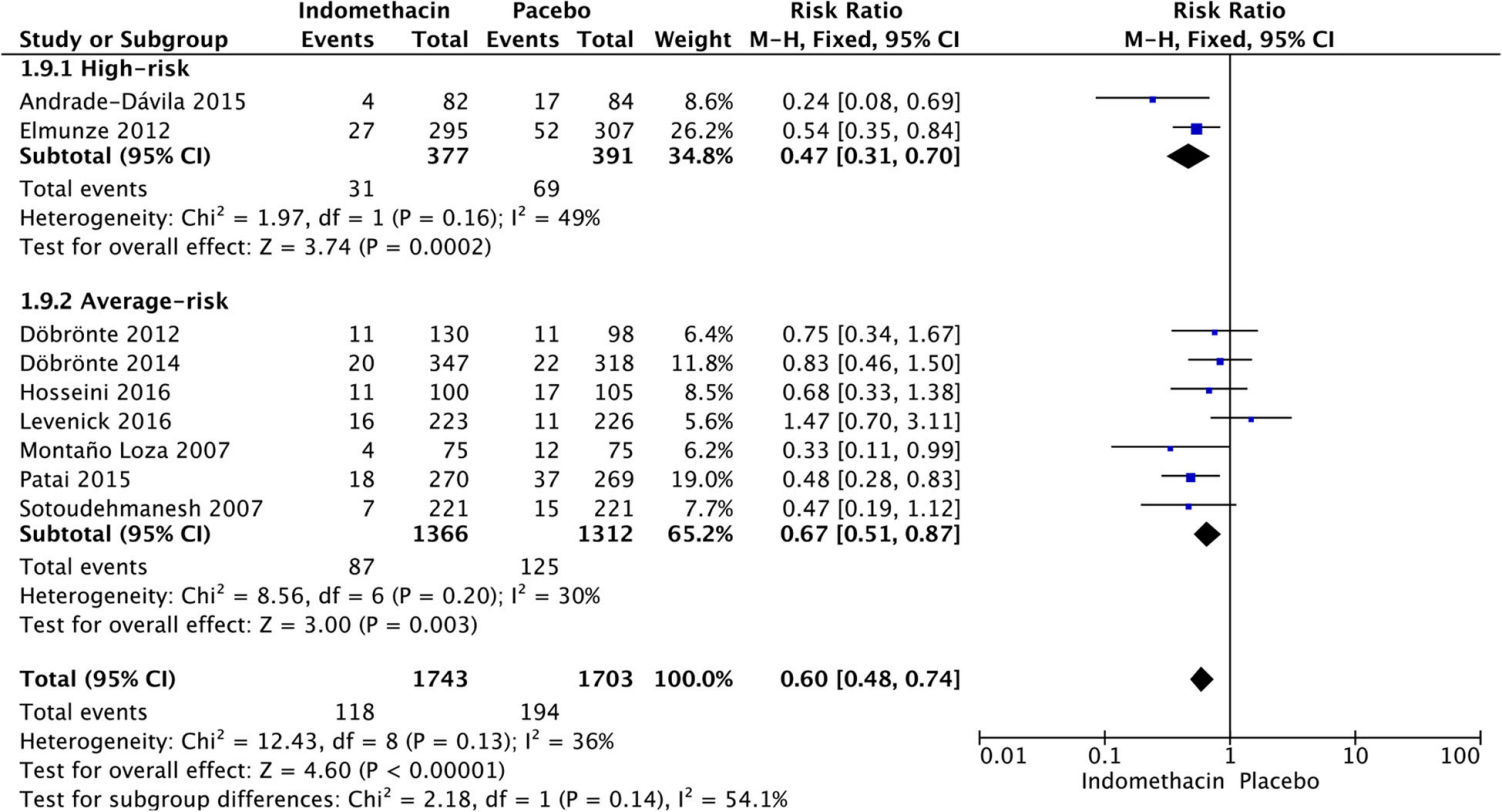
Forest plot of the subgroup meta-analysis of the incidence of PEP by risk in the indomethacin group

**Fig. 12:**
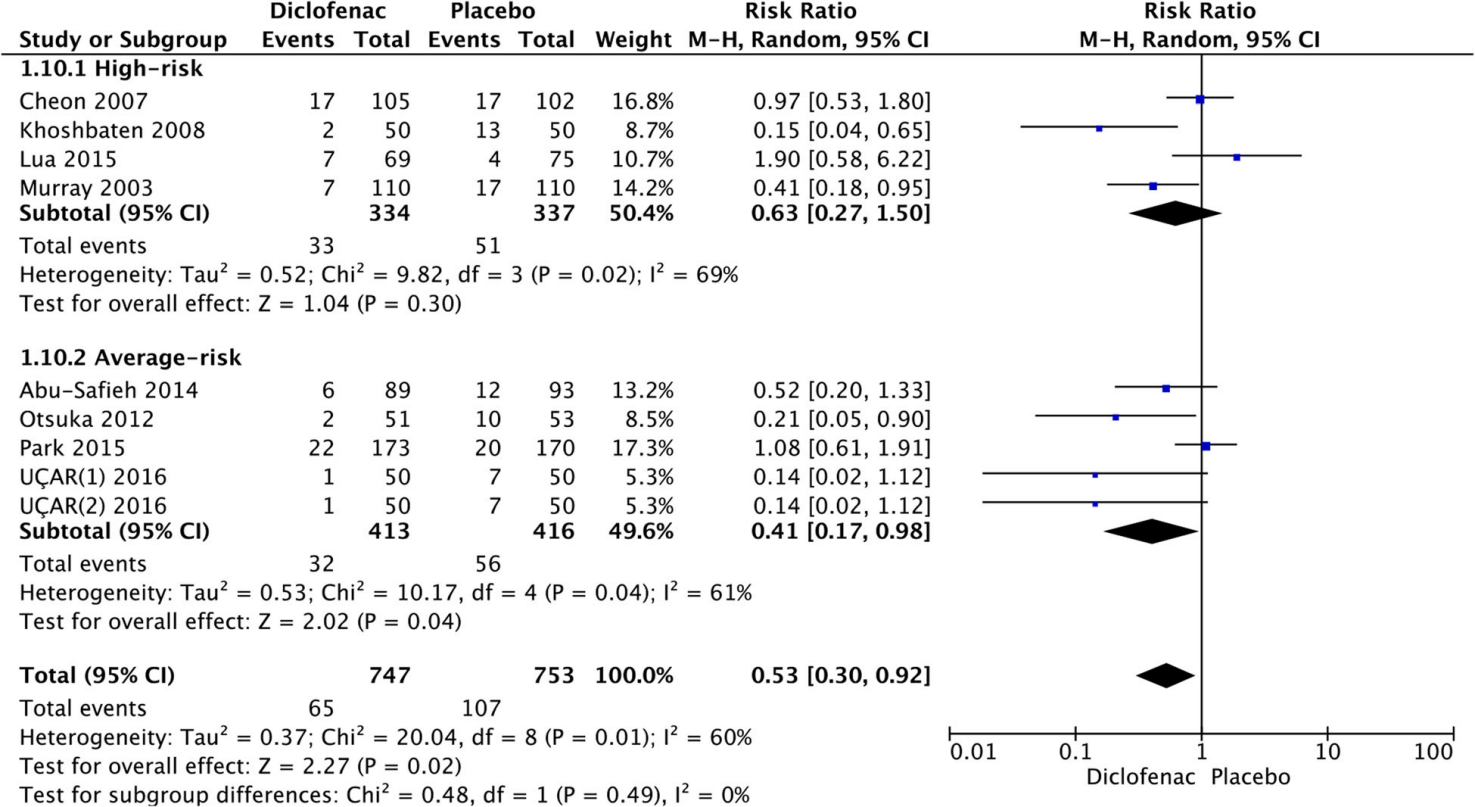
Forest plot of the subgroup meta-analysis of the incidence of PEP by risk in the diclofenac group

After stratification, according to different risk populations, it was noted that NSAIDs were effective in both high-risk (RR = 0.54; 95% CI, 0.41–0.72; *P* < 0.0001) and average-risk patients (RR = 0.61; 95% CI, 0.51–0.72; *P* < 0.00001) (Fig. 10). There was no significant difference between the two groups (*P* = 0.52). Indomethacin was associated with a decrease in the incidence of PEP in the high-risk (RR = 0.47; 95% CI, 0.31–0.70; *P* = 0.0002) and average-risk population (RR = 0.67; 95% CI, 0.51–0.87; *P* = 0.003) (Fig. 11). There was no significant difference between the two groups (*P* = 0.13). Figure 12 shows the risk of PEP among the diclofenac group that included high-risk and average-risk patients for PEP. The estimated pooled relative risks of PEP were 0.63 (95% CI, 0.27–1.50; *P* = 0.30) in high-risk patients and 0.41 (95% CI, 0.17–0.98; *P* = 0.02) in average-risk patients (Fig. 12).

### Publication Bias

**Fig. 13:**
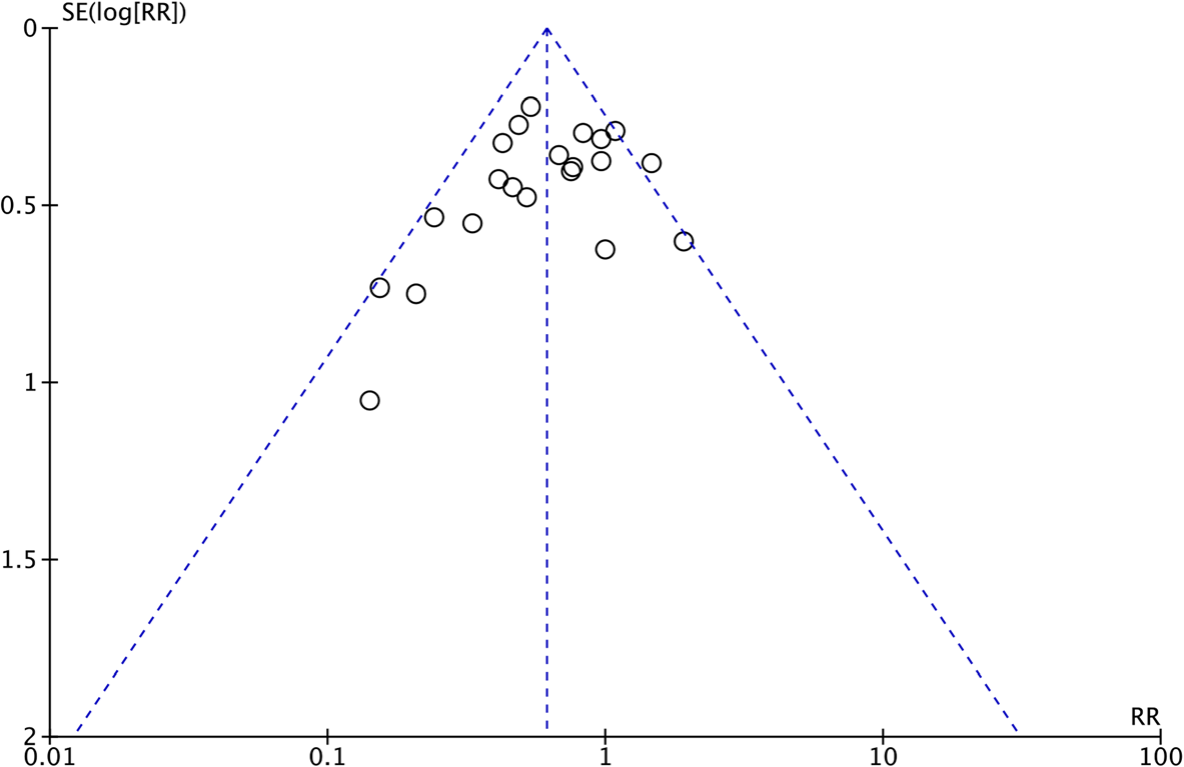
Funnel plot of standard error by log relative risk

A funnel plot analysis was conducted to examine the publication bias. The graphic funnel plot of the 21 studies seemed to be symmetrical, which means that a publication bias is unlikely in this meta-analysis (Fig. 13).

## Discussion

Our meta-analysis showed that the rectal administration of NSAIDs might be the most effective in decreasing the incidence of PEP. Further subgroup analysis showed that indomethacin and diclofenac were able to reduce the incidence of PEP significantly compared with the placebo control. However, no differences were observed between the two drug groups. Rectal was the only effective route of administration. While indomethacin could reduce the incidence of PEP regardless of patients having average or high risk. In contrast, diclofenac could only reduce the incidence of PEP in average-risk patients. In the subgroup analysis of the time of administration, we found that while indomethacin was able to reduce the incidence of PEP when administered before or after ERCP, it seemed to be more effective when administered after ERCP. Meta-analysis showed that pre-ERCP administration of diclofenac could reduce the occurrence of PEP; however, it was unable to do the same when administered post-ERCP.

There have been other research studies on the prevention of PEP administration of NSAIDs recently [23, 36, 37]. Most of the previous studies have confirmed the role of diclofenac and indomethacin in reducing the incidence of PEP. However, unlike previous studies, a recent large-sample retrospective study reported that diclofenac did not reduce the incidence of PEP in low-risk patients compared with the control group [39]. Additionally, in a recent RCT study, Levenick et al. also found that indomethacin did not reduce the incidence of PEP [15]. To the best of our knowledge, to date, no large-scale RCT studies have directly compared indomethacin and diclofenac with the aim of investigating the differences between the two drugs regarding the possibility of preventing PEP. However, only high quality head- to-head RCTs are required in the future. After inclusion of the latest RCT studies, our meta-analysis showed that the incidence of PEP in the indomethacin and diclofenac groups was significantly lower than that in the control group, but there was no significant difference between the two groups alone. No study has compared rectal indomethacin and diclofenac in a head-to-head trial to see if there is any difference in the efficacy between these 2 agents. Previous meta-analysis showed that diclofenac was more effective than other NSAIDs in reducing the incidence of PEP [40, 41]. However, one of the studies described only the rectal route of administration [40], and the other included the study included the rectal 、intravous、intramuscular and oral administration [41]. In contrast, the present study included only comprehensive and recent RCT studies. The influence of a single study on the overall meta-analysis estimate was investigated by omitting one study at a time, and this omission did not have a significant impact on the analysis. However, in all meta-analyses, including the present meta-analysis, the sample of the diclofenac group was relatively small, and the route of administration was different, which could make the results confusing to some extent. Some scholars have suggested that, although there is a certain similarity in the chemical structure, indomethacin is the most potent among all NSAIDs in inhibiting the degree of inflammatory response [42] . Makela et al. found that indomethacin is a better PLA_2_ inhibitor compared with diclofenac [42]. Then, indomethacin would theoretically be better for PEP prevention than diclofenac. However, there is no published study directly comparing the effect of different NSAIDs by rectal administration. Therefore, the difference of indomethacin and diclofenac in the prevention of PEP still needed further study.

Previous studies of different RCTs were different with regard to the time of administration. The meta-analysis included preoperative, intraoperative, postoperative, and preoperative combined with postoperative. The results showed that NSAIDs administered pre-ERCP may be more effective than those administered post-ERCP. This result is similar to the conclusion of a meta-analysis in 2017 that incorporates 16 studies [40]. However, there are different voices on the ideal time of administration [43–46]. Analysis of the causes may be related to the study population, types of NSAIDs, route of administration, and sample of studies included. This meta-analysis analyzes the time of administration of indomethacin and diclofenac, respectively for the first time. Further studies showed that incidence of PEP was lower in the group administered with indomethacin preoperatively and postoperatively than that in the control group and indomethacin appears to be more effective when administered after ERCP. A large multicenter study in 2016 showed better results in preoperative administration by comparing postoperative administration in unselected patients and postoperative administration in high-risk patients, but this study was inadequate for high-risk patients after surgery. Interestingly, in this meta-analysis, the two studies of indomethacin administration involved all high-risk patients. Whether this phenomenon will have an impact on the outcome still warrants more research. Wan et al.*.* showed that preoperative administration of indomethacin was better than postoperative administration [47], but in this study, the authors combined the intraoperative administration of Levenick et al. and postoperative administration [15]. The studies of Inamdar et al. and Sethi et al. showed no difference between the preoperative and postoperative groups [45, 46], but the above studies included eight and seven studies, respectively. The subgroup analysis of diclofenac showed that preoperative diclofenac administration could reduce the incidence of PEP effectively, while the incidence of PEP in the postoperative and control groups did not reveal any difference. The study of Patai et al.*.* showed that there was no differences between before and closely after ERCP in the use of diclofenac or indomethacin [46]. This meta-analysis has not analyzes the time of administration of indomethacin and diclofenac, respectively. However, there is still little research on diclofenac, and further studies are needed to determine the optimal dosing time of diclofenac or indomathacin. Thus, more targeted investigation is needed for determining a specific opportunistic time.

NSAID administration in the prevention of PEP of a specific population is also controversial. The definition of risk of ERCP were varied in the included studies. However, we accepted the original author’s classifications. In this study, we found no difference in the incidence of PEP between average-risk and high-risk patients. A similar conclusion has been drawn in the studies of Shen et al.*..* [48] and Patai et al [46]. This conclusion provides the basis for the recommendations of the ESGE and Japanese Society of Hepato-Biliary-Pancreatic Surgery in its guidelines for the prevention of PEP. Further analysis showed that the effect of indomethacin on the prevention of PEP was not different between average-risk and high-risk patients. In the meta-analysis of Inamdar et al., the authors suggest that indomethacin is effective in high-risk patients and may not be effective for average-risk patients, but only eight studies were included in the analysis, and the authors believe that the study of Patai et al [31]. was for high-risk populations [49]. Elmunzer et al. showed that indomethacin was more effective in high-risk patients, but 82.3% of patients with sphincter of Oddi Dysfunction(SOD) were likely to have an effect in this study [50]. The subgroup meta-analysis of diclofenac group showed that the incidence of PEP had no difference between the high-risk and control groups but was significantly lower in the average-risk group compared with that in the control group. In the previous meta-analysis, there is currently no subgroup analysis of the population of administered with diclofenac. The difference between indomethacin and diclofenac may be related to their differences in inhibition of phospholipase A _2_ [42]. However, in the current RCT and meta-analyses, the definition of PEP risk factors and inclusion criteria are different. Therefore, the above conclusions may be very questionable.

NSAIDs currently have four common routes of administration, including intravenous, intramuscular, oral, and rectal. Of the 21 studies included, 15 studies used the rectal route. Interestingly, so far, a non-rectal administration study concluded that NSAIDs did not reduce the incidence of PEP. Analysis of the causes may be related to the pharmacokinetics of NSAIDs. Rectal administration maximizes drug bioavailability and faster absorption, rapid concentration of drugs, and early suppression of inflammatory responses in pancreatitis. But more clear mechanisms need to be further studied.

However, this study had the following limitations: (1) As the quality of the literature was different, this may have caused some heterogeneity in our study and influenced the conclusion. The heterogeneous definitions of PEP, risk of PEP and indications for ERCP may add potential bias to the results. Also, the heterogeneous of patient (benign or malignant) may cause bias. The criteria of severity for PEP were different in previous studies. (2) This was a meta-analysis at the study level, and confounding factors at the patient level could not be properly assessed and incorporated into the analysis. Therefore, an additional multicenter and large sample of high-quality RCTs is needed to compare the effects of the different routes, dosage, and time of administration on the incidence of PEP.

## Conclusions

Although there are some limitations in this study, we believe that the rectal administration of indomethacin or diclofenac can effectively reduce the incidence of PEP based on our meta-analysis of 21 RCT studies. However, different drug types, their specific time and route of administration, and appropriate population should be considered. More high quality head-to-head RCTs are required.

## Abbreviations

CI: Confidence interval
ERCP: Endoscopic retrograde cholangiopancreatography
NSAIDs: Nonsteroidal anti-inflammatory drugs
PEP: Post-ERCP pancreatitis
RCTs: Randomized controlled trials
RR: Relative Risk
SOD: Sphincter of Oddi dysfunction
